# Treatment Outcomes From a Specialist Model for Treating Tobacco Use Disorder in a Medical Center

**DOI:** 10.1097/MD.0000000000001903

**Published:** 2015-11-06

**Authors:** Michael V. Burke, Jon O. Ebbert, Darrell R. Schroeder, David D. McFadden, J. Taylor Hays

**Affiliations:** From the Nicotine Dependence Center, Department of Internal Medicine (MVB, JOE, DDM, JTH) and Division of Biomedical Statistics and Informatics, Department of Health Sciences Research, Mayo Clinic, Rochester, MN (DRS).

## Abstract

Cigarette smoking causes premature mortality and multiple morbidity; stop smoking improves health. Higher rates of smoking cessation can be achieved through more intensive treatment, consisting of medication and extended counseling of patients, but there are challenges to integrating these interventions into healthcare delivery systems. A care model using a master-level counselor trained as a tobacco treatment specialist (TTS) to deliver behavioral intervention, teamed with a supervising physician/prescriber, affords an opportunity to integrate more intensive tobacco dependence treatment into hospitals, clinics, and other medical systems. This article analyzes treatment outcomes and predictors of abstinence for cigarette smokers being treated using the TTS-physician team in a large outpatient clinic over a 7-year period.

This is an observational study of a large cohort of cigarette smokers treated for tobacco dependence at a medical center. Patients referred by the primary healthcare team for a TTS consult received a standard assessment and personalized treatment planning guided by a workbook. Medication and behavioral plans were developed collaboratively with each patient. Six months after the initial assessment, a telephone call was made to ascertain a 7-day period of self-reported abstinence. The univariate association of each baseline patient characteristic with self-reported tobacco abstinence at 6 months was evaluated using the chi-squared test. In addition, a multiple logistic regression analysis was performed with self-reported tobacco abstinence as the dependent variable and all baseline characteristics included as explanatory variables.

Over a period of 7 years (2005–2011), 6824 cigarette smokers who provided general research authorization were seen for treatment. The 6-month self-reported abstinence rate was 28.1% (95% confidence interval: 27.7–30.1). The patients most likely to report abstinence were less dependent, more motivated to quit, and did not have a past year diagnosis of depression or alcoholism.

Predictable patient characteristics such as level of dependence did predict abstinence, but all patient groups achieved comparable abstinence outcomes. While this study has limitations inherent in a single-center retrospective cohort study, it does suggest that the TTS model is an effective way to integrate more intensive tobacco dependence treatment into outpatient settings.

## INTRODUCTION

Worldwide mortality from tobacco use is expected to double to 10 million deaths per year in the next few decades in the absence of effective action. Cigarette smokers who are between the ages of 30 and 69 have 2 to 3 times the rate of mortality when compared with similar people who have never smoked.^1,2^ Smoking cessation is effective in reducing mortality from coronary heart disease, cerebrovascular disease, COPD, and tobacco-caused cancers; as well as improving health outcomes for surgical, diabetic, and mental health patients.^1,3–6^

There are effective clinical interventions to help people stop smoking. To translate these interventions into practice, guidelines recommend that all patients be systematically screened for tobacco use and that those who use tobacco be provided evidence-based treatment, which consists of both pharmacotherapy and behavioral counseling.^7^ Brief counseling is effective, and particular clinical communication strategies seem to improve the impact of counseling^8,9^; however, more counseling time spent with patients result in better outcomes.^10^ Treatment plans that combine more intensive counseling with individualized medication plans that can include combinations and extended use of medications are associated with still higher smoking abstinence rates.^7^

There have been challenges to implementing more intensive treatment into clinical care. Since the guidelines were first introduced in 1996, and aided by other changes in the healthcare system there has been widespread implementation of some recommendations.^11–13^ Screening of all patients for tobacco use is now a requirement in most healthcare systems. Tobacco quitlines are widely available and can be used to provide more intensive counseling and comprehensive treatment. Some quitlines also provide medication to their participants, or medication may be offered as an adjunct to physician services. However, quitlines often suffer from not being integrated into the healthcare delivery system.^14^ Therefore, improvement is greatly needed, especially regarding delivering more intensive treatment to patients in coordination with the healthcare team.^15,16^

A model that can be integrated into larger medical settings and hospitals is 1 in which a tobacco treatment specialist (TTS) works under the supervision of a prescribing clinician to provide a comprehensive assessment, treatment plan, and behavioral counseling to patients who are being seen in the practice. A TTS is usually an allied health professional with specific training in treating tobacco dependency. Mayo Clinic requires a TTS to have at least a Master's degree in a counseling specialty and maintains a TTS certification. In the United States (US), many TTSs receive their training through a program accredited by the Council for Tobacco Treatment Training Programs, which requires that the educational content is consistent with Clinical Practice Guidelines and competency standards developed by a job task analysis and a panel of experts.^17^ Evidence suggests that a TTS can be more effective than a healthcare provider who fits tobacco into other provider duties.^18^

In the United Kingdom (UK), specialty tobacco treatment clinics are well-integrated into the National Health Service and have demonstrated impressive quit rates.^10,19^ In the US, in-person specialist interventions provided in community settings, or peripherally connected to medical settings, have been shown to be effective; a number of studies have described factors that predict successful outcomes in specialist clinics.^20–24^ Tobacco dependence treatment is now classified as an essential health benefit in the Affordable Care Act and improvements in reimbursement could expand this type of service, enabling better integration of intensive treatment for tobacco dependency into clinical care the delivery.^25^

As a case study of a model for integrating treatment into clinical care in the US, we conducted an analysis of self-reported 6-month smoking abstinence for cigarette smokers who received treatment from a TTS in an outpatient tobacco treatment program at Mayo Clinic in Rochester, MN. We analyzed data on patient demographics, measures of dependence, co-occurrence of psychiatric and medical conditions, and treatment characteristics to describe outcomes and predictors of abstinence for cigarette smokers seen in the clinic over a 7-year period.

## METHODS

### Study Setting

The Nicotine Dependence Center (NDC) provides behavioral counseling and pharmacotherapy for medical outpatients referred by a Mayo Clinic clinician. Patients who are identified as a tobacco user may be referred to the NDC by their medical team, but patients can self-refer. In lieu of referring to the NDC, the medical team might counsel the patient, provide medication, make a referral to a telephone quitline, or not address tobacco with their patient.

All participants provided written general research authorization for this Mayo Clinic Institutional Review Board-approved human subjects study. During our research period, a total of 16,597 unique patients were seen for tobacco dependency by an NDC TTS. We cannot determine how many tobacco users were seen at Mayo Clinic during the study period, but if we assume a 20% smoking rate among all outpatients, we can estimate that the NDC treated about 2% to 3% of Mayo Clinic Rochester patients who use tobacco.

### Procedures

A physician supervises all NDC consultations. The supervising physician is 1 of 4 physicians affiliated with the NDC who share supervisory responsibility, usually rotating weekly. The NDC physician is distinct from the referring physician or other healthcare provider who is affiliated with the patient's primary medical team. The TTS conducts an assessment, formulates a treatment plan with the patient, and provides counseling, education, and clinical follow-up. The NDC supervising physician is available for consultation as needed and to prescribe medications for the patient after reviewing the electronic medical record created by the TTS.

Seven TTSs were employed during the study period, with about 4.5 full-time equivalents at any 1 time. Initial consultations with a TTS were 45 to 60 min in length and include an assessment of current tobacco use, past quit attempts, dependence severity with the Fagerström Test for Nicotine Dependence,^26^ readiness and motivation to stop smoking, and medical and psychiatric comorbidity. The TTS counselor incorporated information gathered from that assessment to collaboratively develop an individually tailored treatment plan that consists of cognitive and behavioral strategies and pharmacological treatment. An interactive patient treatment manual “My Path to a Smoke Free Future,” developed specifically for use with patients seen at Mayo Clinic NDC was used to standardize the educational and counseling content, and chart reviews, monthly case conferences, and peer observation were used to standardize the counseling process. Language translation services were available when needed.

Counseling was provided on topics such as craving management, stress reduction, physical activity, and other practical behavioral skills. Motivational interviewing^27^ skills were consistently employed throughout a consult to create momentum for quitting and to actively engage the patient in treatment planning. Physicians provided oversight through a preapproved medication guideline that informs the discussion with the patient. The supervising physician prescribed a tailored pharmacotherapy based upon communication from the TTS through the electronic health record. Follow-up by telephone or in person for 3 to 4 sessions of 10 to 15 min each was attempted for all patients who made a quit attempt. The schedule for follow-up was determined by patient need and availability, and varied from patient to patient.

Current tobacco use status was ascertained by systematic telephone contact from an office receptionist to the patient's designated phone number at 6 months after the initial TTS counseling session. Self-reported 7-day point-prevalence abstinence was obtained by asking “Have you taken any tobacco, even a puff, in the past 7 days?”^28^ No biochemical confirmation or collateral verification of tobacco abstinence was sought. Three attempts were made to contact each patient during business hours and some weekends. Any patient who was not contacted after 3 attempts or who refused to answer telephone follow-up call was categorized as a smoker. Because of database entry procedures, we were unable to distinguish specific patients who self-reported use of tobacco from those who were adjudicated as smoking because of failure to contact or refusal to answer. Usually slightly more than 60% were successfully contacted.

### Statistical Analysis

Patients who were seen as outpatients and who smoked cigarettes and used no other forms of tobacco were included in the analyses so that study of those in this cohort who use other tobacco products is being analyzed and submitted for publication separately. SAS stat software was used to compute the analysis. The univariate association of each baseline patient characteristic with self-reported tobacco abstinence at 6 months was evaluated using the chi-squared test. In addition, a multiple logistic regression analysis was performed with self-reported tobacco abstinence as the dependent variable and all baseline characteristics included as explanatory variables. For the multivariable analysis, missing values of explanatory variables were imputed using multiple imputations with flexible additive imputation models. Findings from the multivariable logistic regression analysis are summarized using the odds ratio (OR) and corresponding 95% confidence interval (95% CI) with ORs >1.0 indicating an increased likelihood of abstinence.

## RESULTS

During the 7-year study period (1/1/2005–12/31/2011), a total of 6824 unique outpatients who used cigarettes as their only tobacco product and who had provided general research authorization were included in the cohort. Studies of hospital patients and residential patients from an overlapping time period have been published elsewhere.^29,30^ Of these 6824 cigarette smokers, 1917 (28.1%; 95% CI: 27.7–30.1) reported 7-day point prevalence abstinence at the 6-month follow-up. Table 1  summarizes patient demographic data and baseline characteristics associated with 6-month abstinence.

**TABLE 1:**
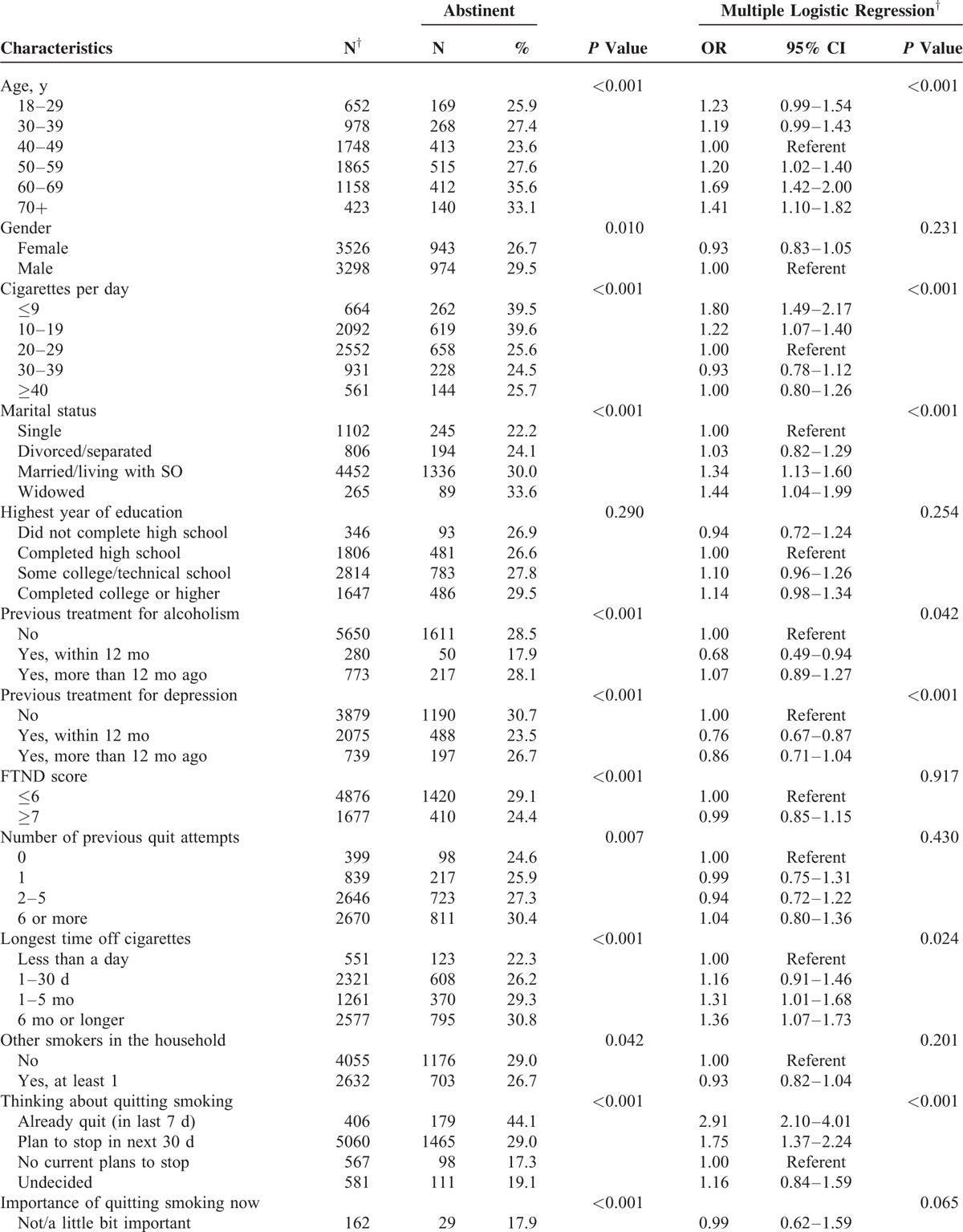
Characteristics Associated With Abstinence at 6 Months Among 6824 Outpatient Cigarette Smokers^∗^

**TABLE 1 (Continued):**
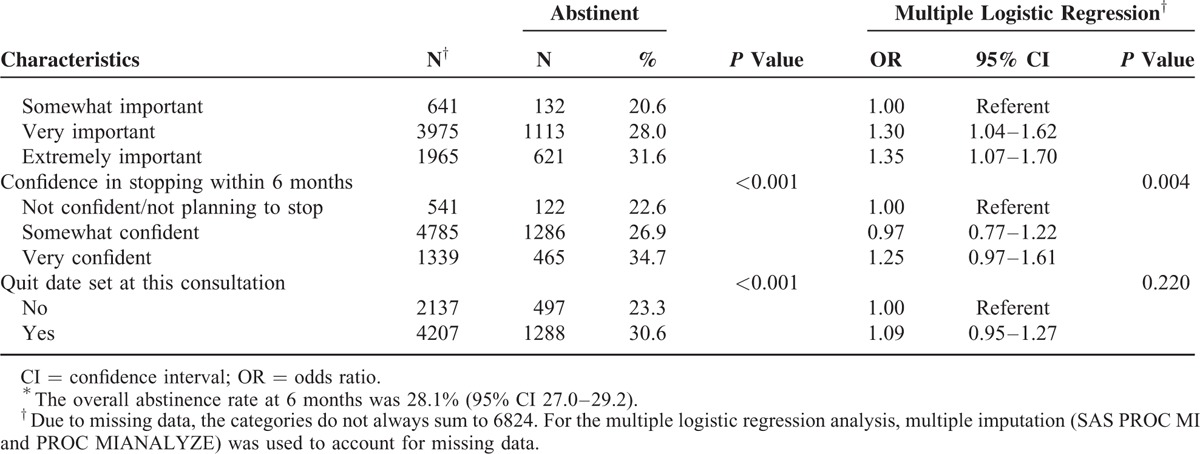
Characteristics Associated With Abstinence at 6 Months Among 6824 Outpatient Cigarette Smokers^∗^

Patients who had stopped smoking within 7 days before their intake, or who were planning to stop within 30 days were significantly more likely to be abstinent than patients without plans to stop. Patients who smoked fewer than 20 cigarettes per day at intake were more likely to report abstinence at the 6-month follow-up compared with patients who smoked more than 20 per day. Those who stated that it was extremely important for them to stop, or that they were very confident they would stop were also more likely to report abstinence. Patients who were treated for alcoholism or depression within the past 12 months were less likely to report abstinence, and patients who were 50 years of age or older were more likely to be abstinent than those 49 and younger.

## DISCUSSION

In our study of cigarette smokers being treated by a TTS in a large medical clinic, we found overall self-reported 6-month abstinence rates of 28%. This compares favorably to results obtained in clinical pharmacotherapy trials,^31,32^ and it is comparable to other studies of clinical populations. Hughes et al^20^ observed that 18% of the patients seen at free state-funded clinics in local hospitals reported abstinence at 12 months after initial contact; however, only 45% of the patients were successfully contacted. Foulds et al^22^ observed that 31.3% of patients reported 6-month abstinence from tobacco in a study of similar patients in a free community setting. The setting did differ from our study in that it is not integrated into a medical clinic. Sheffer et al^24^ found 21% of patients seen at rural health clinics reported abstinence at a 12-month follow-up. In the UK, TTSs and clinics are integrated throughout the different health distracts of the National Health Service, and Kotz and colleagues were able to use population studies to demonstrate that the most effective treatment being provided for tobacco dependence was specialist behavioral support combined with prescription pharmacotherapy. This specialist delivery model in the UK is similar to the TTS and physician/prescriber model described in our study.^10,19^

In our sample, we found positive associations with 6-month smoking abstinence among a wide range of demographic variables. Age was a significant predictor of abstinence, with people who were 50 years of age or older (OR 1.67; *P* < 0.001) more likely to be abstinent at the 6-month follow-up. Smoking fewer than 20 cigarettes per day, or having had a prior quit attempt of 6 months or longer, predicted abstinence. Motivation to quit smoking as measured by willingness to set a quit date, and endorsement that it was “extremely important” to stop smoking, also predicted abstinence. Recent treatment for alcohol or depression, within the past 12 months, was a negative predictor of abstinence at the 6-month follow-up. Previous research has observed that abstaining from smoking is less likely for patients who are more dependent, or for patients who have co-occurring problems with alcohol or depression.^33,34^ While motivation to quit smoking and evidence of taking early actions in the quitting process were predictors of successful quitting, relatively high quit rates were found even among patients who are more dependent, less motivated, or with co-occurring depression and alcoholism.

This study has the inherent limitations of a study analyzing referred patients from clinical practice. An intent-to-treat analysis was used to calculate abstinence rates, but the clinical database did not allow us to count drop outs separately from those who reported continued smoking, which limited the analysis. Patients were not randomized, there was no control intervention, no biochemical confirmation of abstinence was used to validate self-reported abstinence, and the number and frequency of follow-up sessions were not known. We recognize that self-reporting without biochemical confirmation may overestimate abstinence rates, but this seems to be less of a problem when a patient knows that follow-up is part of a treatment plan. Biochemically verified abstinence is usually <5% lower than self-reports.^35^

Our study also has several strengths. It illustrates a model for intensive treatment for tobacco dependence that can be integrated into larger healthcare systems. We describe a large cohort of patients who are at various levels of motivation to quit tobacco and who have typical demographic and comorbid features when compared with other smoking populations. Our results provide a conservative estimate of tobacco abstinence since we counted all patients for whom 6-month follow-up was unavailable as still smoking. We have systematically collected comprehensive patient data and followed up on all of our patients using a standardized protocol. Our population is roughly similar in socioeconomic status to the state of Minnesota, using highest grade as a proxy. The percentage of subjects in this study who completed college or higher is 25%, and the percentage that completed high school is 94.8% compared with 31.4% and 94.0% of Minnesota residents, respectively.^36^

We observed that the physician-supervised TTS model is effective for patients of varied demographics, motivational stages, levels of dependence, and comorbid conditions. Our model could be widely adopted to improve progress in eradicating tobacco use and the resulting morbidity and premature mortality caused by smoking. Tobacco dependence treatment is classified as an essential health benefit within the Affordable Care Act. TTS services can be billed in some settings “incident-to” a physician referral using the model described in this article. Some states, like Minnesota, have designated the TTS as a physician-extender to make for more cost-effective use of professional resources. While further studies are required to compare brief treatment or quitline referral outcomes, cost effectiveness and treatment delivery in a clinical setting, clinics and hospitals can provide services with reasonable quit rates to their patients by investing in TTS training to enable staff members to provide effective evidence-based interventions of higher intensity within the context of a specific healthcare delivery system.
